# Association of beta blockers and mortality in adults with septic shock: systematic review and meta-analysis of randomized clinical trial

**DOI:** 10.3389/fmed.2024.1448573

**Published:** 2024-09-24

**Authors:** Gustavo Adolfo Vásquez-Tirado, Claudia Vanessa Quispe-Castañeda, Edinson Dante Meregildo-Rodríguez, María Cuadra-Campos, Niler Manuel Segura-Plasencia, Yessenia Katherin Arbayza-Avalos, Hugo Nelson Alva-Guarniz, Wilson Marcial Guzmán-Aguilar, Petterson Zavaleta-Alaya

**Affiliations:** ^1^Facultad de Medicina Humana, Universidad Privada Antenor Orrego, Trujillo, Peru; ^2^Escuela de Medicina, Universidad César Vallejo, Trujillo, Peru; ^3^Unidad de Cuidados Intensivos, Hospital Regional Docente de Trujillo, Trujillo, Peru

**Keywords:** adrenergic beta-antagonists, septic shock, mortality, systematic review, meta-analysis

## Abstract

**Introduction:**

Septic shock still entails significant morbidity and mortality, with the heart being affected due to catecholamine overexpression and direct injury from sepsis. Therefore, the effect of β-blocking the receptors to improve performance is promising when attempting to reverse tachycardia and reduce mortality.

**Methods:**

We conducted a comprehensive search across five databases for studies published up to 28 January 2024, using a PICO strategy. Ten studies were identified for quantitative analysis and included in our meta-analysis.

**Results:**

Our meta-analysis evaluated 28-day in-hospital mortality risk across nine randomized controlled trials (RCTs) involving a total of 1,121 adults with septic shock. We found an association between β-blocker use and reduced overall mortality (OR 0.57; 95% CI 0.34–0.98; *I*^2^: 56%). This effect was significant in the esmolol subgroup (OR 0.47; 95% CI 0.26–0.82; *I*^2^: 32%), but not in the landiolol subgroup (OR 0.98; 95% CI 0.0–1,284.5; *I*^2^: 72%). Additionally, the intervention group shows a significant reduction in HR and lactate levels, as well as an increase in stroke volume index (SVI).

**Conclusion:**

In adults with septic shock, β-blockers are associated with a reduction in 28-day in-hospital mortality, a benefit primarily observed with esmolol rather than landiolol. Furthermore, improvements in heart rate (HR) control, lactate levels, and SVI were noted. However, these findings should be interpreted with caution, and further high-quality RCTs comparing different β-blockers are necessary to better elucidate these effects.

**Systematic review registration:**

https://www.crd.york.ac.uk/prospero/, identifier CRD42024513610.

## 1 Introduction

Septic shock (SSh) remains a major condition associated with severe morbidity and mortality. The first-line treatment for SSh includes fluid resuscitation and the administration of vasopressors, alongside infection source control through eradication or antibiotic therapy (1, 2). The physiological response to SSh can lead to deleterious cardiac effects, with the heart being affected in approximately 50% of cases, either due to sepsis itself or excessive catecholamines exposure, such as from sympathetic stimulation. Tachycardia is a poor prognostic indicator in SSh patients (3–5).

It has been postulated that tachycardia, in the context of SSh, further impairs ventricular filling and, thereby reducing the stroke volume index (SVI) and subsequently cardiac output (CO). This is where the role of β-blockers (βb) comes into play; they have been proposed to mitigate these effects, with reports indicating reductions in mortality, lower inflammatory and infectious parameters, and improvements of cardiac, hemodynamic and perfusion markers (5–8).

While others systematic reviews and meta-analyses (SR-Ms) have evaluated the effect of βb on mortality, existing studies have methodological and reporting deficiencies (9–13). These include the incorporation of duplicate studies or those with incorrect intervention groups, failure to use the RoB2 for risk of bias assessment, and the omission of recent RCTs involving landiolol, which have reported no association between βb use and mortality.

Therefore, our SR-Ms aims to evaluate RCTs that incorporate both esmolol and landiolol to assess their association with mortality in patients with SSh. This focus is particularly relevant given that recent high-quality RCTs have independently demonstrated that landiolol use does not reduce mortality.

## 2 Materials and methods

### 2.1 Search strategy

Our systematic review adheres to the methodological standards outlined in the Cochrane Handbook for Systematic Reviews (14), PRISMA guidelines (15), and AMSTAR 2 criteria (16). The study protocol was registered in PROSPERO (CRD42024513610). We conducted a comprehensive search of major databases, including MEDLINE (PubMed), Scopus, EMBASE, Web of Science, Science Direct, and the Cochrane Library. Our search strategy incorporated both controlled vocabulary (e.g., MeSH, Emtree) and free terms, combined with Boolean operators, to implement our PICOS strategy (Population: adult patients with septic shock; Intervention: use of beta-blockers; Comparator: standard treatment; Outcome: 28-day in-hospital mortality; Study design: Randomized clinical trials). Keywords were carefully selected to encompass relevant exposures (“beta blockers” OR “landiolol” OR “esmolol”) and the principal outcome (“In-hospital mortality”). Secondary outcomes were “heart rate,” “stroke volume index,” “lactate,” “norepinephrine doses,” and “length of hospital stay.” A detailed outline of the search strategy is provided in the Supplementary Table 1A).

**FIGURE 1:**
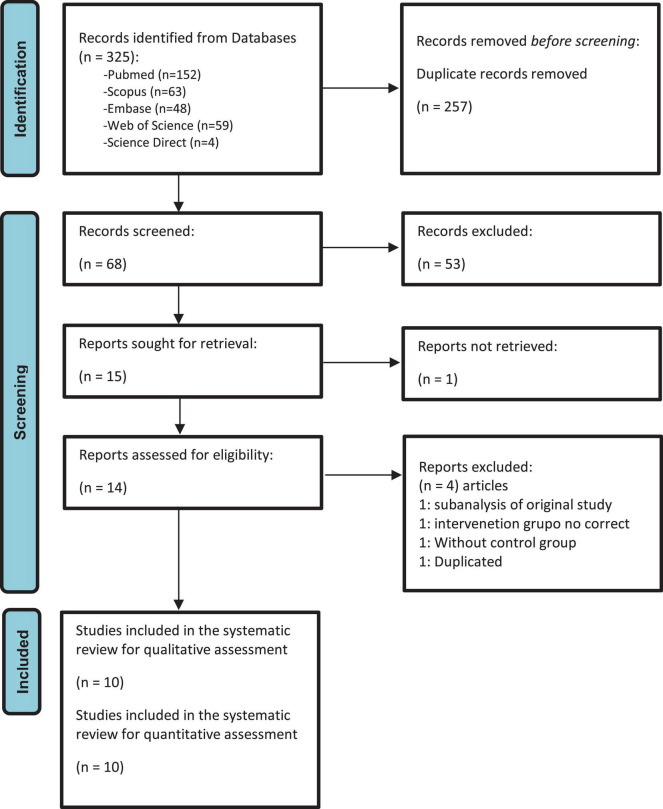
PRISMA 2020 flow diagram of the selection process of the primary studies included.

All articles identified during the primary and secondary screenings were cataloged using Zotero^®^ 6.0.15. After removing duplicates, the documents were transferred to the Rayyan^®^ tool. Here, two authors (GAVT and CVQC) independently screened titles and abstracts in a blinded manner. Studies were selected by mutual agreement, with a third researcher (EDMR) resolving any disagreements. The selected papers underwent a thorough full-text review to determine their eligibility. Furthermore, reference lists and citations of the included publications were manually examined to further enhance the identification of relevant studies. For clarity, the selection process is illustrated in Figure 1. Excluded studies are shown in Supplementary Table 1B.

### 2.2 Selection criteria

We included only RCTs that assessed the use of βb in patients with SSh and their association with mortality as the primary outcome. The studies enrolled adult patients (≥ 18 years old), regardless of the infectious focus. We considered articles published up to 28 January 2024, without restrictions on language or publication date. We excluded case reports, case series, cross-sectional studies, cohort studies, case-control studies and duplicates.

### 2.3 Outcomes

The primary outcome was 28-day in-hospital mortality. Secondary outcomes included heart rate (HR), SVI, lactate, norepinephrine (NE) doses, and length of hospital stay (LoS).

### 2.4 Data extraction

Two independent researchers collected and extracted relevant information from each included study using a standardized, blinded spreadsheet. This information included authors’ names, country and year of publication, clinical and epidemiological characteristics of the population, number of participants and cases, measures of association, confounding factors, and outcomes. For dichotomous variables, we calculated odds ratios (OR) with 95% confidence intervals (CI 95%) based on the events in exposed and non-exposed groups. Missing data were reported when appropriate. For continuous variables, we gathered the mean and standard deviation (SD) for both exposed and non-exposed groups. When studies presented these variables as medians and interquartile ranges (IQR), we converted them to means and SDs using appropriate methods (17, 18).

### 2.5 Statistical analysis

We employed the Inverse Variance (IV) method (19) in the meta-analysis to combine adjusted odds ratios (ORs) and their 95% confidence intervals (CIs). This analysis was performed using R^®^ 4.2.226 software. Forest plots were used to summarize the quantitative synthesis, utilizing the *meta* library, and the *metabin* (20) (for dichotomous variables) and *metacont* (20) (for continuous variables) functions. The IV method with Restricted Maximum Likelihood (REML) for tau (2) was applied. Heterogeneity among studies was assessed using Cochran’s Q test and Higgins I^2^ statistic. In cases of statistically significant heterogeneity (I^2^ statistics > 40%), a random effects model with Hartung-Knapp (HK) adjustment was employed. Sensitivity analysis was conducted using Influence Analysis with the *InfluenceAnalysis* function, and Graphic Display of Heterogeneity (GOSH) with the *gosh.diagnostics* function (21). Additionally, due to the variability in the measures of association for continuous variables across studies, their inclusion in the meta-analysis was deemed inappropriate.

### 2.6 Quality assessment

We assessed the potential risk of bias using the Risk of Bias tool version 2 (RoB2) (22). Additionally, we employed a funnel plot and conducted Egger’s test to investigate publication bias (23). If publication bias was detected, we would determine its impact on the meta-analyzed outcome and consider addressing it using the *Trim and Fill* function (24). Two researchers (GAVT and CVQC) independently evaluated the certainty of the evidence (CoE) for each outcome based on the Grading of Recommendations Assessment, Development, and Evaluation (GRADE) criteria (25, 26). Any discrepancies between reviewers were resolved through discussion with the lead researcher (EDMR).

## 3 Results

### 3.1 Search results and study characteristics

Ten studies were included for qualitative synthesis and meta-analysis (27–36). All studies were RCTs conducted across various countries and continents, including Turkey, Italy, USA, UK, Japan, China, and Egypt, involving a total of 1,121 patients. Among these, 142 out of 394 patients (36%) in the βb group and 189 out of 396 patients (47%) in the control group died. The follow-up period for mortality extended up to 28 days of hospitalization, as not all studies provided follow-up data beyond this period.

Of the included studies, eight used esmolol, and two used landiolol as the βb (30, 31). Both esmolol and landiolol are cardioselective ultra-short-acting β-blockets.

All studies enrolled adult patients with SSh, primarily focusing on pneumonia, followed by abdominal infections. The βb infusion protocol in the intervention group was administered after a stabilization period in both the intervention and control groups, guided by a 24-h intravenous injection protocol. During this period, variables such as HR, SVI, and vasoactive drugs doses were measured, among others.

Additional demographic characteristics of the study population are detailed in Table 1.

**TABLE 1:** General characteristics of included studies.

References	Study design	Participants	Intervention	Outcome	Values
Bingül et al. (27); Turkish.	RCT	A total of 52 patients were evaluated, of which 27 were in the experimental group (βb use) and 25 were in the control group. The primary sources of infection in both groups were pneumonia-induced sepsis and abdominal sepsis. The βb group had an average age of 46.6 years and an APACHE II score of 19.7, which were lower compared to the control group, which had an average age of 55.5 years and an APACHE II score of 20.5.	Esmolol	Primary outcome: In-hospital mortality Secondary outcomes: NE dosage, lactate levels, LoS	Mortality: 6/27 (intervention group) and 7/25 (control group) NE dosage: 0.03 ± 0.06 and 0.06 ± 0.17 LoS: 21.0 ± 19.0 vs. 21.1 ± 19.3 Lactate: 1.47 ± 0.83 vs. 1.92 ± 1.56 Values are for the intervention group and control group, respectively.
Morelli et al. (28); Italia.	RCT	This is an open-label phase 2 RCT, where the primary outcome was achieving HR < 95 beats per minute. A total of 336 patients were enrolled, with 182 being excluded, leaving 77 patients in both the intervention and control groups. Males predominated in both groups. The main sources of infection were pneumonia and peritonitis. The control group had a higher median age (69 vs. 66 years) and a higher SAPS II score (57 vs. 52 points).	Esmolol	Primary outcome: HR < 95 beats per minute Secondary outcomes: 28-day in-hospital mortality, NE dossage, oxygenation index, safety	Mortality: 38/77 (intervention group) and 62/77 (control group) SVI: 34.25 ± 3.8 vs. 30.25 ± 3.75 NE dosage: 0.46 ± 0.28 vs. 0.42 ± 0.26 LoS: 19.0 ± 4.6 vs. 15.0 ± 5.2 Lactate: 1.60 ± 0.40 vs. 1.93 ± 0.58 Values are for the intervention group and control group, respectively.
Cocchi et al. (29); EEUU	RCT	This is a double-blind RCT conducted in 2 hospital centers. Of the 1,122 eligible patients, 1,080 were excluded, leaving 42 patients studied, 22 in the intervention group and 20 in the control group. The origin of sepsis was 33% pneumonia and 30% abdominal infections.	Esmolol	Primary outcome: Hemodynamic profile, NE dosage Secondary outcomes: Mortality, LoS, days on MV, lactate	Mortality: 6/18 (intervention group) and 8/22 (control group) NE: 0.24 ± 0.19 vs. 0.16 ± 0.17 Lactate: 1.90 ± 0.40 vs. 2.10 ± 0.58 LoS: 12.7 ± 5.5 vs. 15.5 ± 6.9 HR: 90.5 ± 7.5 vs. 95.5 ± 5.8 Values are for the intervention group and control group, respectively.
Whitehouse et al. (30); UK	RCT	This is an open-label RCT conducted in 40 ICUs. The main sources of infection were lung and abdominal. The mean age was higher in the exposure group (55.9) compared to the control group (55.3) without a significant difference. The intervention group had a higher SOFA score than the control group (8.8 vs. 8.1). A total of 126 adults were enrolled, with a mean age of 55.6 years, with 63 patients in both the intervention and control groups.	Landiolol	Primary outcome: SOFA score Secondary outcomes: 28-day in-hospital mortality, number of adverse events	Mortality: 23/63 (intervention group) and 16/63 (control group) LoS: 21.3 ± 31.7 vs. 19.6 ± 19.3 Lactate: 3.30 ± 3.10 vs. 2.50 ± 1.60 HR: 92.4 ± 10.4 vs. 98.6 ± 12.2 NE doses: 0.34 ± 0.33 vs. 0.24 ± 0.23 Values are for the intervention group and control group, respectively.
Kakihana et al. (31). Japan.	RCT	This open-label RCT involved 54 hospitals in Japan. A total of 151 patients were enrolled, with 76 in the experimental group and 75 in the control group. The experimental group had a mean age of 67.8 years and an APACHE II score of 22.7, while the control group had a mean age of 66.4 years and an APACHE II score of 23.1. Respiratory infections were the most common (30%), followed by abdominal and urinary tract infections.	Landiolol	Primary outcome: HR control < 94 beats per minute Secondary outcomes: 28-day in-hospital mortality, hospital-free days, lactate, pH, PO2/FiO2	Mortality: 9/75 (intervention group) and 15/75 (control group)
Liu et al. (32). China	RCT	A total of 100 patients with SSh and tachycardia at admission were enrolled, excluding those with prior beta-blocker use, LVEF < 40%, severe valvulopathy, pregnancy, or AV block. Males predominated in both groups, with an average age of 58 years in the intervention group and 57 years in the control group. The average APACHE II score was 18.8 in the intervention group and 19.1 in the control group.	Esmolol	Primary outcome: 28-day in-hospital mortality Secondary outcomes: LoS, lactate levels, HR, NE dosage	Mortality: 31/50 (intervention group) and 34/50 (control group) LoS: 13.3 ± 4.9 vs. 10.8 ± 3.8 Lactato: 1.23 ± 0.25 vs. 2.28 ± 0.75 HR: 103 ± 19 vs. 98 ± 17 NE dosis: 0.17 vs. 0.21 Values are for the intervention group and control group, respectively.
Wang et al. (33). China	RCT	A total of 60 patients were enrolled over 2 years, with 30 patients in both the intervention and control groups. Patients with SSh were included, excluding those with concomitant MI, COPD, or heart failure. The intervention group had a higher average age (67.2 vs. 62.5 years) and APACHE II score (18.4 vs. 15.7) compared to the control group.	Esmolol	Primary outcome: HR control < 90 beats per minute or a reduction of at least 20% from baseline Secondary outcomes: 28-day in-hospital mortality, CI, lactate levels, SVI	Mortality: 9/30 (intervention group) and 11/30 (control group) HR: 86.4 ± 12.1 vs. 97.2 ± 22.6 IC: 3.7 ± 0.99 vs. 3.09 ± 0.88 Lactate: 2.29 ± 1.6 vs. 2.5 ± 2.2 SVI: 38.3 ± 10.1 vs. 31.9 ± 13.2 Values are for the intervention group and control group, respectively.
Xinqiang et al. (34). China	RCT	A total of 48 patients with SSh were enrolled over 2 years in a single-center, double-blind, randomized study.	Esmolol	Primary outcomes: 28-day in-hospital mortality, LoS	Mortality: 6/24 (intervention group) and 15/24 (control group) LoS: 13.75 ± 8.68 vs. 21.7 ± 6.06 SVI: 39.9 ± 2.2 vs. 36.8 ± 1.7 HR: 84.4 ± 3.5 vs. 111.2 ± 7.0 Lactate: 2.8 ± 0.3 vs. 3.4 ± 0.3
Yang et al. (35). China	RCT	A total of 41 adult patients with SSh were enrolled to evaluate the cardioprotective effect of beta-blockers. Patients with heart failure, arrhythmias, or COPD were excluded. The control group had a higher age and APACHE II score (55 years and 21.3 points) compared to the intervention group (51 years and 20.1 points). There were 21 men and 20 women.	Esmolol	Primary outcome: Hemodynamic effects: HR, CI, SVI	No mortality dates. IC: 3.3 ± 0.7 vs. 4.0 ± 0.9 HR: 91 ± 7 vs. 108 ± 14 SVI: 39.0 ± 6.2 vs. 38.0 ± 5.7 Values are for the intervention group and control group, respectively.
Gadallah et al. (36). Egypt	RCT	A total of 60 patients aged 18 to 60 years with SSh were enrolled, excluding those with contraindications for βb use or requiring inotropes. The control group had a lower APACHE II score compared to the intervention group (23.5 vs. 24.2 points) and a younger average age (56.4 years) compared to the intervention group (58.3 years).	Esmolol	Primary outcome: 28-day in-hospital mortality Secondary outcomes: LoS, lactate levels, HR control	Mortality: 14/30 (intervention group) and 21/30 (control group) LoS: 13.2 ± 3.3 vs. 16.2 ± 3.2 Lactate: 3.4 ± 0.59 vs. 3.6 ± 0.8 HR: 88.2 ± 5.8 vs. 105.7 ± 6.9 Values are for the intervention group and control group, respectively.

βb, beta-blockers; SSh, septic shock; CI, cardiac index; HR, heart rate; SVI, stroke volume index; NE, norepinephrine; LoS, length of hospitalization days.

### 3.2 Risk of bias in studies

Of the nine studies included, five had a low risk of bias (29, 30, 32, 33, 36), two had a some concerns of risk of bias (28, 35) and two studies had a high risk of bias (27, 31). For further details, see Table 2.

**TABLE 2:** Risk of bias of the included studies using Risk of bias tool version 2 (RoB2) of Cochrane.

Unique ID	Experimental	Comparator	Outcome	D1	D2	D3	D4	D5	Overall
Morelli et al. (28)	Esmolol	Standard treatment	Mortalidad	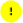	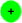	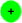	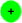	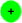	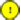
Xinqiang et al. (34)	Esmolol	Standard therapy	Mortalidad	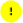	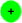	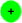	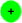	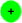	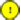
Liu et al. (32)	Esmolol	Standard treatment	Mortalidad	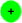	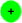	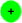	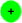	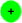	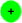
Bingül et al. (27)	Esmolol	Standard therapy	Mortalidad	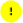	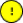	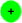	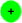	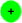	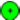
Gadallah et al. (36)	Esmolol	Standard treatment	Mortalidad	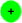	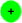	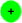	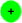	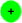	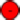
Kakihana et al. (31)	Landiolol	Standard treatment	Mortalidad	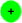	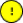	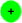	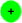	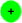	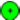
Cocchi et al. (29)	Esmolol	Placebo	Mortalidad	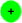	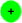	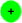	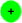	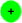	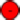
Whitehouse et al. (30)	Landiolol	Standard care	Mortality	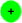	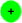	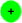	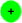	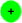	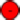
Wang et al. (33)	Esmolol	Placebo	Mortalidad	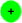	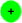	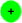	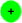	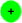	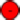

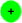
, Low risk. 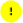, some concerns; 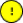, High risk; D1, Randomization process; D2, Deviations from the intended interventions; D3, missing outcome data; D4, measurement of the outcome; D5, selection of the reported result

### 3.3 Beta-blockers and mortality in septic shock

**FIGURE 2:**
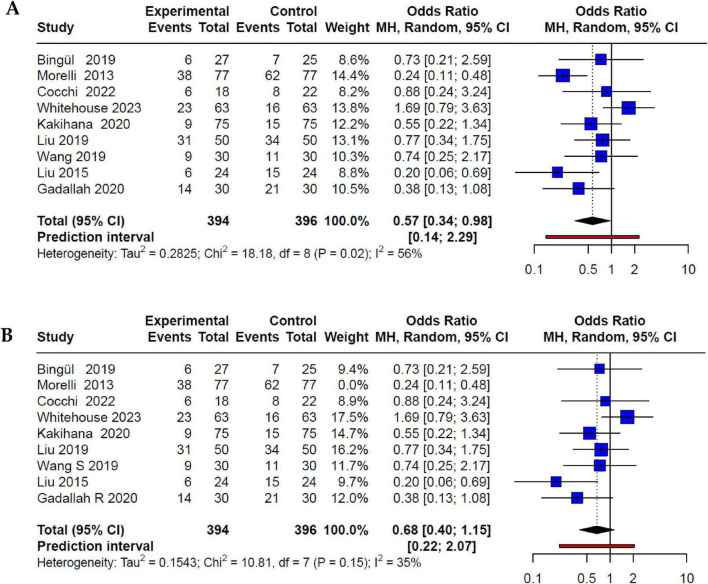
**(A)** Forest plot of beta-blocker use and mortality in adults with SSh. All studies were included in the initial meta-analysis, showing high heterogeneity, which after the detection of outliers through GOSH, Morelli et al. (28) was eliminated. **(B)** Forest plot after exclusion of outliers showing the effect of beta-blocker use and mortality in adults with SSh with acceptable heterogeneity.

Among the ten studies included in the meta-analysis, all except Yang et al. (35) had mortality data for both the intervention (βb) and control groups. The initial meta-analysis revealed a 43% reduction in mortality risk in the intervention group (OR 0.57; 95% CI 0.34–0.98; *p* < 0.05). However, this analysis revealed unacceptably high heterogeneity (I^2^: 56%) and a prediction interval (PI) that did not demonstrate a consistent decrease in the risk of mortality in future studies (95% PI 0.14–2.29) (Figure 2A). In accordance with the Cochrane Systematic Review and Meta-analysis (SR-Ms) manual, given the significant heterogeneity, a detailed evaluation into potential sources of heterogeneity was performed. Subsequent sensitivity analysis employing Influence Analysis and GOSH, identified the study conducted by Morelli et al. (28) as an outlier. Upon its exclusion, the sensitivity analysis demonstrated minimal heterogeneity (I^2^: 35%). However, no significant association between βb use and reduced mortality risk was observed (OR 0.68; 95% CI 0.40–1.15; *p* > 0.05) (Figure 2B).

**FIGURE 3:**
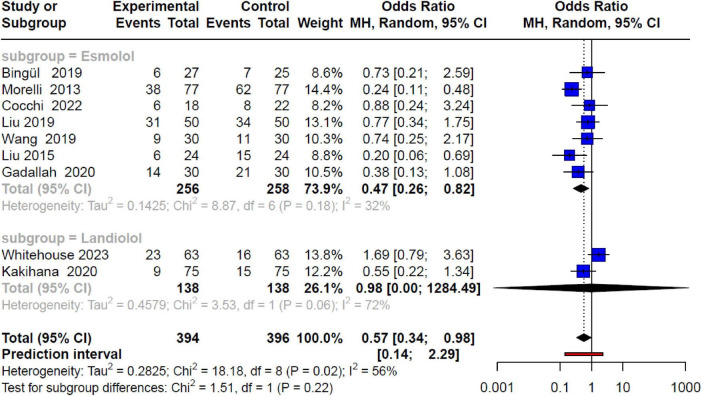
Forest plot by subgroups of beta-blockers (esmolol and landiolol) and mortality in SSh, showing a reduction in risk only in the esmolol subgroup with acceptable heterogeneity, losing significant association in the landiolol subgroup.

Furthermore, subgroup analysis based on the type of βb revealed notable distinctions. Studies employing esmolol exhibited a 53% reduction in mortality risk (OR 0.47; 95% CI 0.26–0.82; *p* < 0.05) with low heterogeneity (I^2^: 32%). In contrast, studies using landiolol did not demonstrate a significant decrease in mortality risk (OR 0.98; 95% CI 0.0–1,284.5; *p* > 0.05), showcasing substantial heterogeneity (I^2^: 72%) (Figure 3).

Finally, it is important to note that when heterogeneity was analyzed by subgroups based on the risk of bias, considering the groups with “some concerns” as high risk, the meta-analysis showed an association in the high-risk (OR 0.35; 95% CI 0.14–0.90; I^2^: 29%) group but lost the association in the low-risk group (OR 0.85; 95% CI 0.42–1.70; I^2^: 27%).

### 3.4 SVI, HR and NE doses like hemodynamic parameters

**FIGURE 4:**
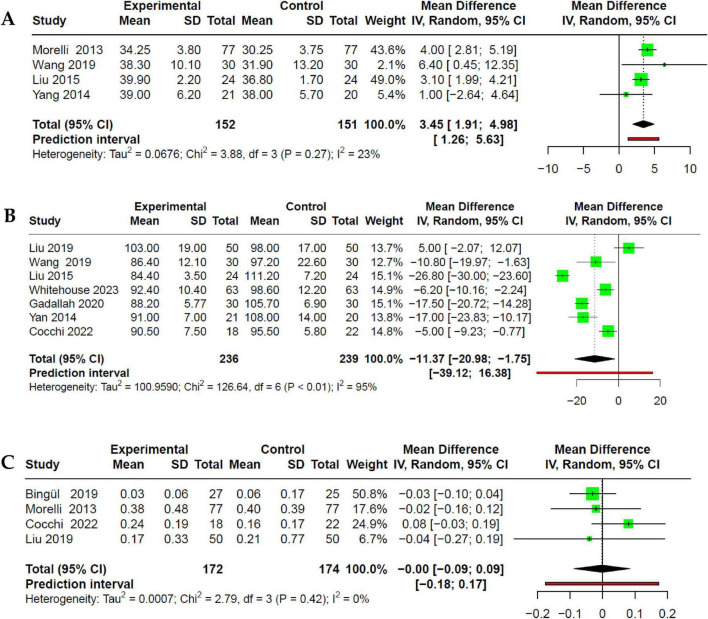
Forest plot of the effect of beta-blocker use in SSh on **(A)** improvement of SVI; **(B)** reduction of HR. **(C)** No effect is evident on the dose of NE used.

Only four RCTs assessed SVI as a measure of hemodynamics (28, 32, 33, 35). In these studies, the use of esmolol was associated with a significant increase of SVI compared to the control group (MD 3.45; 95% CI 1.91–4.98; *p* < 0.05) with low heterogeneity (I^2^: 20%) (Figure 4A). Additionally, among seven studies (29, 30, 32–36), the intervention group exhibited a significant reduction in HR compared to the control group (MD −11.4; 95% CI −20.9 to −1.75; *p* < 0.05). However, this analysis showed high heterogeneity (I^2^: 95%). When assessing heterogeneity through Influence Analysis, Xinqiang et al. (34) behaved as an outlier; but its exclusion, did not substantially decrease heterogeneity (I^2^: 90%). Subgroup analysis based on the type of βb revealed that statistical significance persisted for both the esmolol and landiolol groups (MD −12.24; 95% CI −23.92 to −0.56; *p* < 0.05 and MD −11.37; 95% CI −20.98 to −1.75; *p* < 0.05, respectively), without reducing heterogeneity (I^2^: 95%) (Figure 4B). Finally, when evaluating five RCTs (27–30, 32), we found no association between the use of beta-blockers and NE doses (MD −0.0; 95% CI −0.09 to 0.09; *I*^2^: 0%) (Figure 4C).

### 3.5 Lactate as a marker of perfusion

**FIGURE 5:**
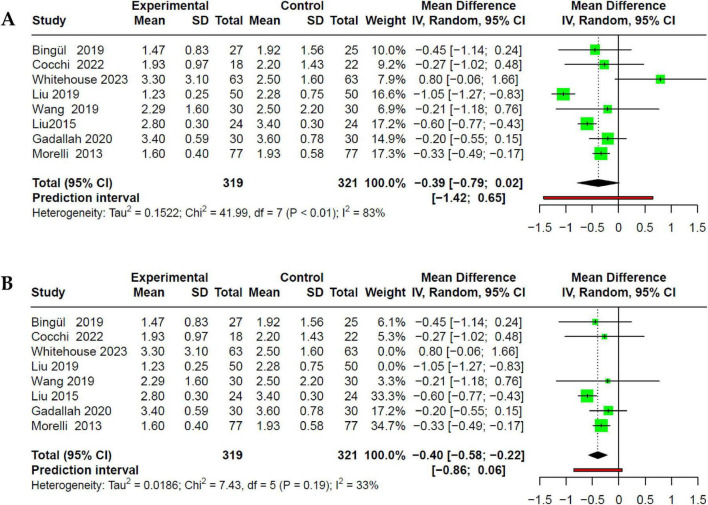
Forest plot of the effect of beta-blocker use in SSh on serum lactate. **(A)** No association is shown on lactate concentration, but with very high heterogeneity. **(B)** Sensitivity analysis, where, through Influence Analysis, Liu et al. (32) and Whitehouse et al. (30) are excluded for being outliers.

Data on lactate values were extracted from eight studies (25–28, 30–32, 34). Lactate levels were serially measured in both intervention and control groups at baseline and every 24 h. We reported lactate values in both groups at 72 h, during which both groups had completed the initial stabilization phase and βb administration. Of the eight studies, only Whitehouse et al. (30) evaluated landiolol, while the remaining studies assessed esmolol. Although the intervention group exhibited a decrease in lactate values at 72 h, this reduction was not statistically significant compared to the control group (MD −0.39; 95% CI −0.79 to 0.02; *p* > 0.05) with high heterogeneity (I^2^: 83%) (Figure 5A). Heterogeneity assessment through Influence Analysis identified Whitehouse et al. (30) and Liu et al. (32) as outliers. Excluding these studies revealed a significant reduction in lactate levels in the intervention group (MD −0.40; 95% CI −0.58 to −0.22; *p* < 0.05), and a decrease in heterogeneity (I^2^: 33%) (Figure 5B).

### 3.6 LoS

**FIGURE 6:**
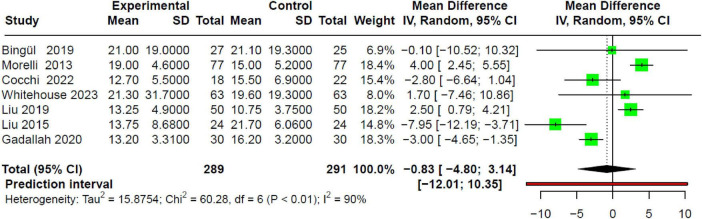
Forest plot of the effect of beta-blocker use in SSh on LoS, showing no association.

Seven studies that evaluated the length of hospital stay (27–30, 32, 34, 36) showed a very slight reduction in duration in the intervention group, but without statistical significance compared to the control group (MD −0.83; 95% CI −4.8 to 3.14; *p* > 0.05), with high heterogeneity (I^2^: 90%) (Figure 6).

### 3.7 GRADE assessment and funnel plot

**FIGURE 7:**
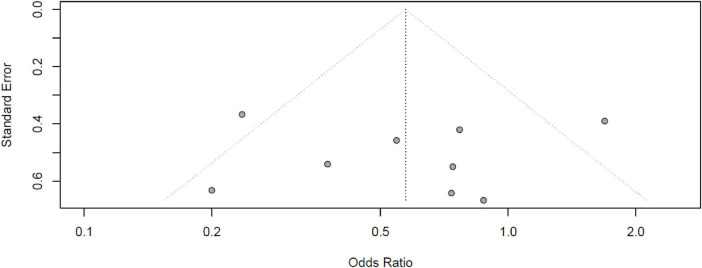
Funnel plot of the included studies in the meta-analysis on the effect of beta-blocker use and mortality in SSh with Egger’s test: –0.35; 95% CI –5.4 to 4.7; *p* > 0.1. No publication bias is evident.

We used GRADE to assess the certainty of evidence (CoE) regarding the association between βb use and mortality in SSh. We found no publication bias [Egger’s test (23): −0.35; 95% CI −5.4 to 4.7; *p* > 0.1] (Figure 7). Table 3 shows a total reduction of 43% in overall mortality risk with βb use. Subgroup analysis indicates that esmolol is linked to a 53% reduction in mortality risk, with moderate certainty of evidence for this effect. Conversely, landiolol does not demonstrate a significant reduction in mortality risk in SSh, with a low certainty of evidence.

**TABLE 3:** Certainty of evidence through GRADE.

Outcomes	No of participants (studies) Follow-up	Certainty of the evidence (GRADE)	Relative effect (95% CI)	Anticipated absolute effects	
				Risk with standard	Risk difference with betablock
Overall mortality	790 (9 RCTs)	⊕⊕⊕○ Moderate	OR 0.57 (0.35 to 0.94)	477 per 1,000	135 fewer per 1,000 (235 fewer to 15 fewer)
Mortality (esmolol)	514 (7 RCTs)	⊕⊕⊕○ Moderate	OR 0.46 (0.29 to 0.74)	612 per 1,000	192 fewer per 1,000 (298 fewer to 73 fewer)
Mortality (landiolol)	276 (2 RCTs)	⊕⊕○○ Low	OR 0.98 (0.33 to 2.98)	225 per 1,000	3 fewer per 1,000 (137 fewer to 239 more)

CI, confidence interval; OR, odds ratio. The use of beta-blockers shows a 43% lower mortality risk in adults with septic shock, maintaining this finding when analyzed by subgroups with the use of esmolol (both with a moderate level of certainty), while there is no significant evidence of decreased risk of death with the use of landiolol (with a low level of certainty).

## 4 Discussion

### 4.1 Beta-blockers and mortality risk

Our meta-analysis of nine randomized controlled trials (RCTs) involving a total of 1,121 patients reveals that β-blocker (βb) use in patients with SSh reduces mortality by up to 43% (OR 0.57; 95% CI 0.34–0.98; *I*^2^: 56%). This result includes both esmolol (seven studies) and landiolol (two studies). However, subgroup analysis based on the type of βb revealed that the benefit persists only in the esmolol subgroup (OR 0.47; 95% CI 0.26–0.82; *I*^2^: 32%), while no significant mortality reduction is observed in the landiolol subgroup (OR 0.98; 95% CI 0.0–1,284.5; *I*^2^: 72%).

Profound hemodynamic instability in patients with SSh ultimately leads to multiple organ dysfunction and increased mortality. The potential benefits of βb in various medical conditions suggest their potential use in critically ill patients with SSh, where reflex tachycardia leads to decreased SVI and, consequently, cardiac output (CO). β-blockers can help mitigate this response by controlling or reducing HR, thereby allowing more time for ventricular filling, improving both SVI and CO. This physiological mechanism supports that βb use can reduce inflammatory markers, improve perfusion indicators (such as lactate levels), an enhance hemodynamic variables [such as SVI, CO, and cardiac index (CI)] (6, 8, 37, 38).

In addition to the well-documented role of βb in improving hemodynamic parameters, they are proposed to have a significant function in modulating the dysregulated inflammatory response observed in sepsis. βb attenuate the deleterious cardiac effects of pro-inflammatory cytokines such as IL-1β, IL-6, and TNF-α, which induce an up-regulation of adrenergic receptors, thereby exacerbating hemodynamic instability. The administration of beta-blockers helps to mitigate these effects. Additionally, βb are postulated to play a role in modulating lymphocyte apoptosis driven by the inflammatory processes inherent in sepsis and sepsis-induced coagulopathy (3, 11).

Some SR-Ms evaluate βb, but ours has several strengths over existing research, such as incorporating more recent, higher-quality RCTs from diverse international settings, including the latest studies evaluating landiolol; thereby advancing the current state of knowledge in this field.

Huang et al. (12) in an SR-Ms assessing the efficacy and safety of esmolol in patients with SSh, included fourteen randomized controlled trials (RCTs), the largest number of studies reviewed to date. They focused on 28-day in-hospital mortality as the primary outcome and found that esmolol use was associated with a reduced mortality risk (RR = 0.66, 95% CI = 0.56–0.77, *p* < 0.001; *I*^2^: 0%), based on data from nine RCTs (eight Chinese studies and one Italian). However, methodological aspects of this study need to be addressed, such as using the Jadad scale for risk of bias assessment instead of RoB2. Additionally, the study selection process was not rigorous enough, incorporating an RCT with a different intervention group (milrinone and esmolol), which distorts the average mortality data and other outcomes.

Zhang et al. (10) in an SR-Ms exploring the benefits of esmolol in patients with SSh, analyzed eight RCTs and found that βb use reduces mortality (RR 0.68; 95% CI 0.52–0.88; *I*^2^: 45%). However, the study has methodological limitations, including the inclusion of RCTs with some risk of bias, except for Liu et al. (32). Additionally, like other published SR-Ms, this analysis duplicated a study reported in another journal and included an RCT with an inadequate outcome (combining milrinone and esmolol as the intervention group) (39), potentially altering the overall RR result.

Similarly, Li et al. (9) conducted an SR-Ms analyzing six RCTs in order to assess the prognosis of βb use in patients with SSh. Their meta-analysis of four RCTs reported a mortality reduction of up to 41% in the intervention group (RR 0.59; 95% CI 0.48–0.74; *I*^2^: 0%), consistent with our data. However, the study has methodological shortcomings, as one of the six RCTs had an erroneous intervention group (combining milrinone and esmolol) (39), and another study was an analytical version of Morelli’s original work (40). Moreover, the risk of bias assessment using the Jadad scale, suggest that the quality of the studies’ is moderate to high. However, this assessment might differ if evaluated with RoB2 tool per Cochrane and PRISMA guidelines.

Interestingly, RCTs with esmolol as the intervention do not consistently exhibit high quality according to the RoB2 assessment. In contrast, RCTs evaluating landiolol, which generally demonstrate better methodological quality, fail to show a reduction in mortality. This discrepancy could be attributed to limitations in study designs or specific drug effects, despite esmolol and landiolol being ultra-short-acting βb with similar pharmacokinetic properties. However, Ikeshita et al. (41), in an experimental study in mice, showed that landiolol is more versatile due to slightly greater cardioselectivity and fewer cardiodepressant effects than esmolol, although both drugs exhibit comparable negative chronotropic effects.

Finally, Hasegawa et al. (11) conducted an SR-Ms similar to ours, evaluating the impact of ultra-short-acting βb on mortality in SSh patients. It incorporates six RCTs with esmolol and one RCT with landiolol, concluding that there is a significant reduction in mortality in the experimental group (RR 0.68; 95% CI 0.54–0.85; *I*^2^: 31%). However, like the previous SR-Ms, one study has a different experimental group (milrinone plus esmolol), and it does not evaluate subgroups for the two types of ultra-short-acting βb (esmolol vs. landiolol and mortality effect in each subgroup), which are addressed in our report.

It is important to emphasize that our research is the first to incorporate recent RCTs using landiolol as a βb. We meticulously reviewed several databases and included only those RCTs that address our PICO question. This approach contrasts with other published SR-Ms, which often include studies with duplicates or inappropriate intervention groups, such as those assessing the combination of esmolol with milrinone, which deviates significantly from our PICO question. Additionally, we adhered to Cochrane and PRISMA guidelines for risk of bias assessment using RoB2 tool, rather than relying on less suitable scales. Our analysis also involved a thorough exploration of heterogeneity using Influence Analysis and GOSH, with findings validated through the GRADE approach.

### 4.2 Beta-blockers and hemodynamic parameters

In our results, additionally, βb use in patients with SSh improves hemodynamic parameters, showing an increase in SVI, assessed in four studies (MD 3.45; 95% CI 1.91 to 4.98; *I*^2^: 23%). β-blockers also significantly reduce heart rate (HR) (MD −11.37; 95% CI −20.98 to −1.75; *I*^2^: 95%) and lactate levels (MD −0.30; 95% CI −0.60 to −0.01; *I*^2^: 64%). This effect is maintained only in the esmolol subgroup (MD −0.37; 95% CI −0.55 to −0.18; *I*^2^: 45%), while no significant effect is observed in the landiolol subgroup (MD 0.80; 95% CI −0.06 to 1.66). Furthermore, no association was found between β-blocker use and the dosage of vasoactive agents such as norepinephrine (NE) or LoS.

Although the RCTs used primarily evaluate mortality as their main outcome, they have also assessed hemodynamic parameters, perfusion markers such as lactate, and even inflammatory markers in patients with SSh.

Furthermore, Huang et al. (12) reported that the intervention group experienced significant reductions in HR (SMD −17; 95% CI −2.24 to −1.17; *I*^2^: 90%) and troponin *I* levels (SMD −1.61; 95% CI −2.06 to −1.16; *I*^2^: 69%) but found no significant differences in mean arterial pressure (MAP) (SMD 0.13; 95% CI −0.03 to 0.29; *I*^2^: 0%).

Similarly, Zhang et al. (10) also reported that in six RCTs that the intervention group achieved adequate HR control and reduction at 72 h (DM −1.91; 95% CI −3.23 to −0.60; *I*^2^: 94%). Additionally, this research showed a statistically significant HR reduction from 12 to 72 h of treatment (SMD −1.91; 95% CI −3.23 to −0.60; *I*^2^: 94%), an improvement in CI only at 72 h (SDM −0.40; 95% CI −0.73 to −0.07; *I*^2^: 0%), and reduced troponin *I* levels from 24 to 72 h (SMD −1.62; 95% CI −2.54 to −0.73; *I*^2^: 73%). However, no significant differences were observed in central venous pressure (CVP) (SMD 0.03; 95% CI −0.29 to 0.36; *I*^2^: 0%), SVI (SMD 0.43; 95% CI −0.54 to 1.41; *I*^2^: 88%), lactate levels (SMD −0.40; 95% CI −0.93 to 0.12; *I*^2^: 80%), and MAP (SMD 0.07; 95% CI −0.25 to 0.39; *I*^2^: 0%).

Additionally, Li et al. (9) the study also found a significant reduction in HR (SMD −2.01; 95% CI −3.03 to −0.98; *I*^2^: 93%) and troponin *I* levels (SMD −19.91; 95% CI −2.39 to −1.43; *I*^2^: 0%), but no improvement between esmolol use and SatVCO2 (SMD 1.87; 95% CI −1.53 to 5.36; *I*^2^: 97%), lactate levels (SMD 0.77; 95% CI −0.66 to 2.21; *I*^2^: 94%), CVP (SMD 0.18; 95% CI −0.02 to 0.39; *I*^2^: 0%), or MAP (SMD 0.18; 95% CI −0.02 to 0.39; *I*^2^: 0%).

### 4.3 Strengths

Our study has numerous strengths. First, we carried out a comprehensive search strategy, covering six essential databases and exclusively RCT studies, allowing a robust epidemiological evaluation of cause and effect. Second, we used a rigorous methodology to conduct our review and meta-analysis, including a thorough quality assessment of studies and a statistical analysis to address heterogeneity. Third, our results are both robust and reliable, as evidenced by our careful assessment of heterogeneity and identification of outliers, with consistent findings across individual studies. Fourth, this is the first SR-Ms of adequate quality that incorporates the most recent RCTs conducted with landiolol.

### 4.4 Limitations

However, it is important to acknowledge some limitations in our study. First, only a few completed studies have explicitly addressed our PICO question; most studies are in Chinese, making them difficult to access. Second, while current studies adhere to the Sepsis-3 definition of SSh, older studies still rely on the SIRS criteria, creating inconsistencies in the data. Finally, there are only two RCTs involving landiolol, and none employ a three-arm design to compare β-blockers, placebo, and both together; which limits our ability to fully elucidate the specific effects of esmolol and to definitively assess landiolol’s impact on mortality in SSh patients.

## 5 Conclusion

Our study suggests that using β-blockers (βb) in patients with SSh can reduce mortality; however, this effect is observed exclusively with esmolol, and there is no group effect since landiolol does not demonstrate similar effects. Consistent with these findings, better HR control is also evidenced, improving hemodynamic parameters such as SVI and enhanced perfusion (reduction in lactate) in the experimental group. However, given the current evidence, these results should be interpreted with caution. To further elucidate the effects of β-blockers, additional RCTs with robust designs are needed, particularly those that evaluate landiolol specifically or employ a three-arm study design comparing both β-blockers to placebo.

## Data Availability

The original contributions presented in this study are included in this article/Supplementary material, further inquiries can be directed to the corresponding author.
