# Supplementary material for: Association of beta blockers and mortality in adults with septic shock: systematic review and meta-analysis of randomized clinical trial
Source: Front Med (Lausanne). 2024 Sep 24;11:1448573. doi: 10.3389/fmed.2024.1448573 (PMC11458450; doi:10.3389/fmed.2024.1448573)
Supplement: Supplementary file 1 [file Table_1.DOCX]

Table S1A- Search strategy

| 1. MEDLINE VIA PUBMED | | Results | Date |
| --- | --- | --- | --- |
| #1 | (((beta blockers) OR (esmolol)) OR (landiolol)) OR (heart rate control) | 497,338 | 28/January/2024 |
| #2 | **septic shock** | 43007 |  |
| #3 | #1 AND #2 (Filters: Clinical Trial, Randomized Controlled Trial) | 152 |  |

| 2. EMBASE | | Results | Date |
| --- | --- | --- | --- |
| #1 | ('beta adrenergic receptor blocking agent'/exp OR 'beta adrenergic receptor blocking agent' OR 'esmolol'/exp OR esmolol OR 'landiolol'/exp OR landiolol OR 'labetalol'/exp OR labetalol) AND ([controlled clinical trial]/lim OR [randomized controlled trial]/lim) | 19652 | 28/January/2024 |
| #2 | ('septic shock'/exp OR 'septic shock') AND ([controlled clinical trial]/lim OR [randomized controlled trial]/lim) | 2983 |  |
| #3 | #1 AND #2 | 48 |  |

| 3. WEB OF SCIENCE | | Results | Date |
| --- | --- | --- | --- |
| #1 | **(TI=((((beta blockers) OR (esmolol)) OR (landiolol)) OR (heart rate control))** | [11731](https://webofscience.upao.elogim.com/wos/woscc/summary/a71cabe6-7984-45f3-9868-6a11dbe95210-73415b19/relevance/1) | 28/January/2024 |
| #2 | **TI=(septic shock)** | 10625 |  |
| #3 | #1 AND #2 | 59 |  |

| 4. SCOPUS | | Results | Date |
| --- | --- | --- | --- |
| #1 | TITLE-ABS-KEY (((beta blockers) OR (esmolol)) OR (landiolol)) OR (heart rate control) | 14962 | 28/January/2024 |
| #2 | TITLE-ABS-KEY ( septic shock) ) | 11098 |  |
| #3 | #1 AND #2 | 63 |  |

| 5. SCIENCE DIRECT | | Results | Date |
| --- | --- | --- | --- |
| #1 | TITLE-ABS-KEY (((beta blockers) OR (esmolol)) OR (landiolol)) OR (heart rate control) | 5377 | 28/January/2024 |
| #2 | TITLE-ABS-KEY (septic AND shock ) | 2376 |  |
| #3 | #1 AND #2 | 4 |  |

Table S1B. Excluded studies for meta-analysis and the reason for their exclusion

| Study | Reason for exclusion |
| --- | --- |
| Nishida O, Kakihana Y, Okajima M, et al. A multicenter randomized controlled study on landiolol for the treatment of sepsis-related tachyarrhythmia: Subanalysis of the JLand 3S study. Crit Care. 2020;24. doi:10.1186/s13054-020-2772-3 | No adecuate data. |
| Wang Z, Wu Q, Nie X, Guo J, Yang C. Combination Therapy with Milrinone and Esmolol for Heart Protection in Patients with Severe Sepsis: A Prospective, Randomized Trial. Clin Drug Invest. 2015;35(11):707-716. doi:10.1007/s40261-015-0325-3 | Intervention group is erroneous. |
| Du W, Wang XT, Long Y, Liu DW. Efficacy and Safety of Esmolol in Treatment of Patients with Septic Shock. Chin Med J (Engl). 2016 Jul 20;129(14):1658-65. doi: 10.4103/0366-6999.185856. PMID: 27411452; PMCID: PMC4960954. | No control group. |
| Brown S, Beesley S, Lanspa M, et al. Esmolol Infusion in Patients with Septic Shock and Tachycardia: A Prospective, Single-Arm Pilot Study. AMERICAN JOURNAL OF RESPIRATORY AND CRITICAL CARE MEDICINE. 2018;197. | Cohort study type |
| Harvey H, Gupta J, Billyard T. Effect of heart rate control with esmolol on haemodynamic and clinical outcomes in patients with septic shock 2C03, 3C00. J Intensive Care Soc. 2014;15(3):262-263. doi:10.1177/175114371401500321 | No RCT |
| Jain T, Mawri S, Shah J, et al. Pre-Hospitalization Use Of Beta-Blockers And Clinical Outcomes In Septic Shock Patients Admitted To Intensive Care Unit. AMERICAN JOURNAL OF RESPIRATORY AND CRITICAL CARE MEDICINE. 2016;193. | No PICO accord |
| Unger M, Morelli A, Singer M, et al. Landiolol in patients with septic shock resident in an intensive care unit (LANDI-SEP): Study protocol for a randomized controlled trial. Trials. 2018;19(1). doi:10.1186/s13063-018-3024-6 | A protocol, no data |
| Wauschkuhn S, Fuchs C, Scheer C, et al. Reduction of 90-day mortality in patients with severe sepsis and septic shock by initiation of oral beta-blocker treatment and continuation of a previous beta-blocker therapy. INFECTION. 2015;43:S56-S56. | No retrieve |
